# Evidence-based orthopedics: One step closer!

**DOI:** 10.4103/0019-5413.73651

**Published:** 2011

**Authors:** Mohit Bhandari, Anil K. Jain

**Affiliations:** *Department of Orthopaedics, McMaster University, Hamilton, Ontario, Canada*; 1 *Prof. of Orthopaedics in University College of Medical Science, Senior Orthopaedic Surgeon, GTB Hospital, Delhi, India*

The balanced application of the evidence (the clinical decision-making) is the central point of practicing evidence-based orthopedics (EBO) and involves, according to evidence-based medicine principles, integration of our clinical expertise and judgment with patients’ perceptions and societal values, and with the best available research evidence.^1^

EBO involves careful attention to the design, statistical analysis, and critical appraisal of clinical research. The delineation between “outcomes” research and “evidence-based medicine” is vague. Since the term evidence-based medicine was coined first at McMaster University, orthopedic surgeons and researchers have adopted their own style of critical appraisal often coined as “evidence-based orthopedics (EBO)” using a clear delineation of relevant clinical questions, a thorough search of the literature relating to the questions, a critical appraisal of available evidence and its applicability to the clinical situation, and a balanced application of the conclusions to the clinical problem.^1^,^2^

Gaining momentum from the global EBM movement, the concepts and ideas attributed to and labelled collectively as EBO have become a part of daily clinical lives, and surgeons increasingly hear about evidence-based guidelines, evidence-based care paths, and evidence-based questions and solutions. The controversy has shifted from whether to implement the new concepts to how to do so sensibly and efficiently, while avoiding potential problems associated with a number of misconceptions about what EBO is and what it is not. The EBO-related concepts of hierarchy of evidence, meta-analyses, confidence intervals, study design are so widespread that health care providers willing to use today’s medical literature with understanding have no choice but to become familiar with principles and methodologies of evidence-based orthopedics.

Understanding the ongoing need to adapt to evidence-based approaches and the success of our 2008 symposium on evidence-based medicine,^1^–^4^ the *Indian Journal of Orthopaedics* is highlighting a new section entitled, “Evidence-Based Orthopaedics: Tips for Clinical Practice” for its readers. This novel section of the journal will publish concise and practical tips to reviewing the orthopedic literature. The aim is, of course, to empower the journal readership with the tools necessary to become efficient in their critical appraisal techniques of the published literature. Simply put, this section will assist readers in generating better evidence as well as understanding the validity of published papers. We use a 3-min checklist approach to allow our readership to critically evaluate the reliability of a given study in a few short minutes. A further enhancement in the Journal includes an “Evidence Scan”, a section aimed to provide an evidence summary of one or two highly important recent publications in our field.

We hope that both additions will serve to engage readers and move the *Indian Journal of Orthopaedics* one step closer to its goal among the top sources of orthopedic evidence.
